# Space resource utilisation: a novel indicator to quantify species competitive ability for light

**DOI:** 10.1038/srep16832

**Published:** 2015-11-23

**Authors:** Pengfei Zhang, Xiaolong Zhou, Junyong Li, Zhi Guo, Guozhen Du

**Affiliations:** 1 State Key Laboratory of Grassland and Agro-Ecosystems, School of Life Sciences, Lanzhou University, Lanzhou, Gansu Province, 730000, P. R. China

## Abstract

Species richness and productivity are two fundamental aspects of ecosystems. As a result, the relationship between species richness and productivity has been widely studied. A series of fertilisation experiments in an alpine meadow on the Tibetan Plateau were performed to study the relationship between species richness and productivity. In this paper, we present a novel indicator, i.e., space resource utilisation (SRU), which is calculated by a volume formula (V_i_  =  h_i_ · S_i_; h_i_ = plant height of species i, S_i_ = quadrat area × percent cover of species i). SRU more fully reflected species competitive ability for light in both horizontal and vertical dimensions compared with plant height and cover. We used this novel indicator to investigate the effects of SRU on the changes in species richness and productivity following fertilisation. We found that the SRU of the community was correlated with increasing productivity and decreasing species richness following fertilisation and was a better predictor of species richness than productivity. The changes in SRU following fertilisation vary among species. These results demonstrate that SRU can be a more useful tool in explaining plant biodiversity loss and predicting the fate of different species than each of height, cover and productivity.

Nutrient enrichment (eutrophication) is considered as one of the primary factors that decreases species richness worldwide^1^,^2^,^3^,^4^. Over the past one hundred years, many grassland experiments have been conducted to study the relationship between species richness and productivity^5^,^6^,^7^,^8^,^9^. The initial conclusion from these studies was that species richness consistently exhibited a unimodal (i.e., increasing then decreasing) relationship or negative correlation with the increase in productivity that resulted from fertilisation^10^,^11^. However, recent meta-analyses have shown different relationships between species richness and productivity, and the generalisation of a hump-shaped patterns has been questioned^8^,^12^,^13^.

Until now, three competition-based hypotheses have been proposed to explain the reduction in species richness that occurs with an increase in nutrient availability resulting from fertilisation^14^,^15^,^16^,^17^. First, the total competition hypothesis predicts that above- and below-ground competition become more important after fertilisation, which leads to mortality and reduces species richness^18^,^19^. Second, the light competition hypothesis predicts that shoot competition causes greater competitive exclusion and mortality compared with root competition when soil resources are abundant^3^,^20^,^21^. Third, the density hypothesis, or community-level thinning, predicts that shaded and small individuals of all species die and are lost from plots randomly^16^,^22^,^23^,^24^. These hypotheses suggest that competition for resources will cause species exclusion following fertilisation; alternatively, species will survive under different nutrient conditions^17^,^25^,^26^. However, each hypothesis emphasises different aspects of competition. For any of the three hypotheses, conflicting results have consistently been obtained from different experiments^3^,^26^. Hence, the present hypotheses and mechanisms are not sufficient or complete.

To better understand the mechanism underlying the decrease in species richness and increase in productivity after fertilisation, a series of field experiments were performed on the Tibetan Plateau^11^,^27^,^28^,^29^. Here we propose a novel indicator and a conceptual model (Fig. 1). In addition to light and nutrients, space is required for plant growth and is the basis of light competition^23^,^30^. We define the space resource utilisation (SRU) as the product of plant height, percent cover and quadrat area, and propose that it can be used as a three-dimensional space resource. The theoretical volume of each species was defined as the space resource utilisation of species (SRUs) and was used to analyse the performance of individual species; the total volume of all the species in each quadrat was defined as the space resource utilisation of the community (SRUc) and was used to study the variation in productivity and species richness.

The model in Fig. 1 reflects the relationships between SRU and species richness in different environments. In unfertilised natural plots, the plant community occupies the entire space resource (R in Fig. 1a), but each species (n_1_, n_2_, n_3_ … n_6_, n_…_) occupies only a portion of R (Fig. 1a). If the functional traits and competition among species do not change following fertilisation, the proportion of R occupied by each species should increase proportionately with the increase in R and therefore the plant community composition (n_1_, n_2_, n_3_ … n_6_, n_…_) should not change (Fig. 1b). However, the proportion of R occupied by each species changed in the actual fertilised environment, resulting in a change in the community composition (Fig. 1c).

Using this indicator and model, the SRU competition hypothesis is proposed here to understand the mechanisms by which fertilisation decreases species richness and increases biomass. SRU reflected the competitive ability in both horizontal and vertical dimensions. At the community level, there were considerable increases in vegetation height and total coverage following fertilisation, which increased SRUc. SRUc was positively correlated with the effective light receiving area, which is directly related to productivity. That is why productivity increased following fertilisation. At the species level, fertilisation increased the SRUs of some species and then increased their utilisation of light, which improved their competitive ability for light. In other species, fertilisation decreased their SRUs and then decreased their utilisation of light, which reduced their competitive ability for light. These effects can lead to a gradual disappearance in species with low competitive ability through competitive exclusion by species with high competitive ability for light^3^,^30^. That is why species richness decreased following fertilisation.

For this study, we address two questions

1. Is SRUc correlated with increasing productivity and decreasing species richness following fertilisation?
2. Is SRUc a better predictor of species richness following fertilisation than productivity?

## Results

### Effects of SRUc on richness and productivity

Above-ground biomass increased significantly (P < 0.05) in response to each of the N5, N10 and N15 levels in both 2012 and 2013, although the differences among N levels were not significant (P > 0.05, Fig. 2a). Species richness decreased significantly at the N15 level (P < 0.05) in 2011, 2012 and 2013 (26, 27 and 22 species, respectively) and the N10 level (25 species, P = 0.002) in 2013, as compared to the control (31, 33 and 35 species in 2011, 2012 and 2013, respectively; Fig. 2b). Above-ground biomass significantly (P < 0.05) increased at all N addition levels in both 2012 and 2013, but species richness decreased significantly at moderate and high N addition levels (N10 and N15) in 2013 and high N addition level (N15) in 2012. Thus, the effect of fertilisation on productivity was observed earlier than the effect on species richness, and the effect of fertilisation on species richness reflected a distinct N-treatment effect (Fig. 2a,b).

Above-ground biomass was not significantly correlated with species richness in either 2012 or 2013 (r = −0.234, P = 0.307 and r = −0.376, P = 0.070, respectively; Fig. 3a). However, there was a significant negative correlation between SRUc and species richness in 2013 (r = −0.518, P = 0.010, Fig. 3c). Despite the significant positive correlation between above-ground biomass and SRUc in both 2012 and 2013 (r = 0.526, P = 0.014 and r = 0.789, P < 0.001, respectively; Fig. 3b), SRUc and above-ground biomass are not equivalent indicators of plant species richness nor do they vary simultaneously (Figs 2 and 3). As expected, SRUc had a positive correlation with productivity and a negative correlation with species richness.

### Effects of SRUs on different species

At the species level, above-ground biomass was more closely correlated with SRUs (r = 0.869, P < 0.001 and r = 0.984, P < 0.001 in 2012 and 2013, respectively; Fig. 4c) than with plant height (r = 0.350, P < 0.001 and r = 0.537, P < 0.001 in 2012 and 2013, respectively; Fig. 4a) or coverage (r = 0.852, P < 0.001 and r = 0.956, P < 0.001 in 2012 and 2013, respectively; Fig. 4b). In the CK treatment, different species had different SRUs values, and the changes in the SRUs values following fertilisation depended on the level of N applied (Table 1, S1, S2). In addition, divergent changes were observed within functional groups, i.e., the SRUs of graminoid species increased, whereas the SRUs of non-leguminous forbs significantly decreased and leguminous forbs almost disappeared from the community after fertilisation (Table 1).

Following fertilisation, *Oxytropis kansuensis*, *Tibetia himalaica*, *Potentilla fragarioides*, and *Euphrasia pectinata* were endangered and threatened (P < 0.05); *Elymus nutans* was the most dominant (P < 0.05); and *Agrostis hugoniana*, *Carex atrofusca* and *Anemone rivularis* were the coexisting species (P > 0.05; Table 1). Therefore, different changes in SRUs gave rise to the different fates after fertilisation.

## Discussion

Plant height and percent cover are frequently used as indicators of plant communities^31^, whereas SRU, which is an aggregative indicator of plant height and percent cover, has not been used. Plant height and percent cover reflected the species competitive ability in the vertical and horizontal dimension, respectively. However, SRUs reflected the competitive ability in both horizontal and vertical dimensions. That is why SRUs is better correlated with biomass compared with height and cover in Fig. 4. Borer *et al.* 2014 studied the role of nutrients and herbivores in grassland plant diversity and reported that nutrient addition resulted in species loss through increased competition for light, especially in productive systems^3^,^32^. At the species level, the disproportionate changes in height and cover following fertilisation have different effects on light competition. SRUs was an aggregative indicator of horizontal and vertical dimensions and therefore can be considered as a driving force intensifying competition for light, which reduced species richness. At the community level, there were considerable increases in vegetation height and coverage (Table S1, S2), which increased SRUc. In addition, SRUc was positively correlated with the effective light receiving area, which is directly related to productivity. Hence, SRUc has a positive correlation with productivity (Fig. 3b).

As shown by Adler *et al.* 2011, productivity is a poor predictor of species richness^8^. Our results support their suggestion that biomass is weakly correlated with species richness (Figs 2 and 3a). in our experimental community, some plants with wispy stems provided a lot of shade but not much biomass, and some plants with large stems provided little shade but much biomass. Therefore, biomass was not a sufficiently good indicator of light competition. SRUc, however, is an aggregate indicator of light competition in both horizontal and vertical dimensions. SRUc was significantly correlated with species richness and is therefore a better predictor of species richness.

**Figure 1:**
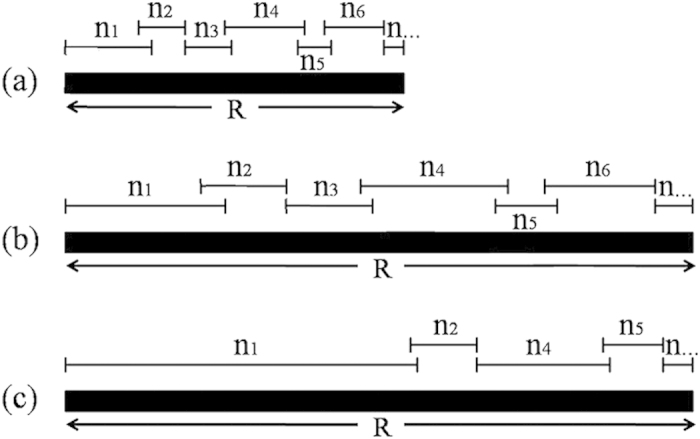
A conceptual model of the relationship between the space resource utilisation and species richness. Each species (n_1_, n_2_, n_3_ … n_6_, n_…_) utilises a portion of the space resource (R) in (**a**) the unfertilised environment, (**b**) the proportionately increased theoretical environment or (**c**) the actual fertilised environment.

**Figure 2:**
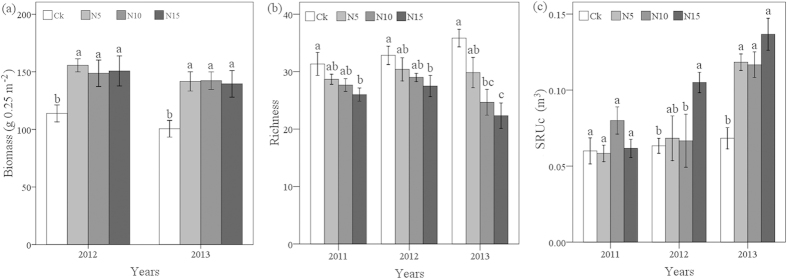
Effects of N addition on (**a**) biomass, (**b**) species richness and (**c**) SRUc (mean ± SE, n = 6). Values with the same letter within a year are not significantly different (p > 0.05).

**Figure 3:**
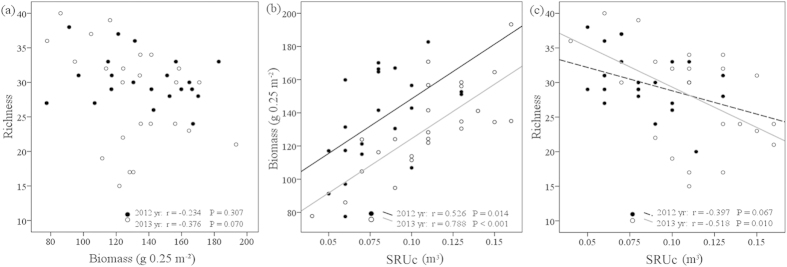
The relationship between (**a**) richness and biomass (**b**) biomass and SRUc (**c**) richness and SRUc. r and p values were estimated from Pearson product-moment correlations.

The conceptual model in Fig. 1 is useful to understand the contrasting effects of SRU on species richness and productivity. The proportion of R occupied by each species (i.e. SRUs) varied following fertilisation, which increased competition for light. Species live in environments that comprise multiple resources^33^. By combining these resources together, the effects of fertilisation on the plant community can be described (Fig. 5). The change in plant height and percent cover following fertilisation varied among species (Tables S1 and S2). These changes directly affected SRUs (Table 1). In addition, SRUs had a positive impact on the utilisation of light^3^,^34^. Hence, the changes in plant height and cover indirectly affect the utilisation of light^35^. These resources collectively affect the community composition (Fig. 5).

Because of different functional traits and competition, species have different requirements for a particular resource^36^,^37^. Species in a community have coexisted for a long time because species with high competitive ability do not exclude others when present in high abundance, and species with low competitive ability can persist even when present in low abundance^38^,^39^. Specifically, SRUs can satisfy the requirements for reproduction as well as growth and survival in the natural community. SRUc increased after fertilisation, while there was variation in SRUs (Table 1). Fertilisation increased SRUs and competition for light by some species. In other species, however, fertilisation decreased their SRUs and ability to compete for light. These effects can lead to a gradual disappearance in species with low competitive ability through competitive exclusion by species with high competitive ability^3^,^23^,^30^. SRUc and SRUs can be used to explain why productivity increases and species richness decreases with the addition of nitrogen.

We present a simple model (Fig. 6) to better demonstrate the different changes of SRUs after fertilisation (Table 1)^33^. Although the SRUs values differed among species within a natural plant community, each of them was greater than the reproduction level (CK in Fig. 6), which ensured these species can coexist in this natural community. After fertilisation, there were three kinds of changes in the utilisation of a resource (black histogram in Fig. 6). First, above the level required for reproduction (A and B in Fig. 6), species could reproduce and coexist until significant changes occurred (e.g. *Elymus nutans*, *Poa crymophila keng*, *Anemone rivularis* in Table 1). Second, between the survival and reproduction levels (C and D in Fig. 6), species could also survive but not reproduce; therefore, they could not flower or produce mature seeds, which resulted in a gradual disappearance of these species (e.g. *Anemone trullifolia* in Table 1). Third, below the survival level (E in Fig. 6), species could not survive, resulting in a rapid disappearance (e.g. *Oxytropis kansuensis* and *Tibetia himalaica* in Table 1).

Similar to the three kinds of changes in SRUs after fertilisation (black histogram in Fig. 6), if the critical values that correspond to the performance of a species (i.e., survival, growth, reproduction) can be quantified in the future, they can be used to predict a species fate earlier than otherwise^40^. First, a long-term experiment is needed to simulate nutrient enrichment (eutrophication). Over this period, the SRUs values and timing of species extinction can be measured. Then, these data can be used to analyse the relationship between the SRUs values and the status of a species. Our results show that the SRUs values of some species decreased gradually until they were extinct (Table 1). Hence, the critical values of SRUs for disappearing species can be confirmed through data analysis in the future. To determine the fate of a species within a habitat, we can calculate the actual SRUs value and compare this value with the critical values that can be confirmed in the future. Before a species fate can be predicted, the condition that the habitat and plant community composition do not change significantly must be satisfied.

In conclusion, by adopting a novel indicator (i.e., SRU) and a conceptual model (Fig. 1), we identified and quantified several key resources of plant communities. In addition, we tested the ability of this indicator to explain the effects of fertilisation on productivity and species richness. Our results suggest that SRU, which is correlated with productivity and species richness, can be a useful tool in explaining the effects of fertilisation and serve as a better predictor of species richness than productivity.

## Methods

### Study area

The experiment was conducted in a relatively flat alpine meadow of the Research Station of the Alpine Meadow and Wetland Ecosystems of Lanzhou University (Azi Branch Station) in Maqu (101°51′E, 33°40′N), Gansu, China. The site is located on the eastern Tibetan Plateau at 3500 m above sea level. The mean monthly temperature ranges from −10 °C in January to 11.7 °C in July, and the mean annual temperature is 1.2 °C, with approximately 270 frost days per year. The annual precipitation (620 mm) measured over the last 35 years falls mainly during the short, cool summer. There are approximately 2580 h of cloud-free solar radiation annually^7^,^11^,^41^. The vegetation in this area, which is categorized as a typical Tibetan alpine meadow, is dominated by *Kobresia* spp. (Cyperaceae), *Elymus nutans*, *Agrostis* spp., *Festuca ovina*, *Poa* spp. (Poaceae), *Anemone rivularis* (Ranunculaceae) and *Saussurea* spp. (Asteraceae)^11^,^27^. Typically, there are 25–40 vascular plant species and 80–140 g above-ground biomass (dry mass) per quadrat (0.25 m^2^)^27^.

### Study design

In early May 2011, sixty 10 × 20 m plots were established at the study site and surrounded by iron wire fence. Twenty-four plots were used for a nitrogen (N) addition experiment, and the remaining plots were used for experiments on phosphorus (P) and nitrogen and phosphorus (N & P) addition. The plots were separated by 1-m buffer strips. The treatments included three levels of N addition (treatment N5 = 5 g N m^−2^ year^−1^; N10 = 10 g N m^−2^ year^−1^; N15 = 15 g N m^−2^ year^−1^) and a control treatment without nitrogen addition (CK). Each treatment was replicated six times. The plots were laid out in a randomized complete block design. Nitrogen was applied as ammonium nitrate (NH_4_NO_3_) and was broadcasted annually by hand in early May. Fertiliser was applied prior to heavy rainfall to avoid the need for irrigation^28^.

### Vegetation and biomass samples

Twenty-two common species, which accounted for 70–90% of the above-ground biomass and coverage, were sampled from the left half of each plot to measure the reproductive allocation^41^. Thirty individuals of three species (*Elymus nutans*, *Kobresia capillifolia* and *Anemone rivularis*) and twelve individuals of the remaining species were sampled from each treatment. Species were sampled at the full-bloom stage, and only the above-ground plant parts were collected. The height of each sample was measured, and samples were separated into vegetative (stem and leaf) and reproductive (flower and fruit) parts to calculate the reproductive allocation. Then, the samples were dried and weighed to the nearest 10^−4^ g.

In mid-August of 2011, 2012 and 2013, vegetation in a 0.5 × 0.5 -m quadrat was harvested from each plot. The quadrat location was randomly selected from the right half of the plot to avoid the influence of previous sampling. Three individuals that appeared more than three times in the quadrat were randomly selected, and their heights were measured. Then, the heights of the remaining individuals were measured. The number of individuals and ramets of clonal species were recorded, and the cover of each species and the entire plant community was estimated. Species with relatively low cover were assigned a value of 0.5%^27^. The above-ground biomass (approximately 2 -cm residue) was clipped in 2012 and 2013. The harvested biomass was separated into individual species, and the samples were dried at 80 °C for 48 h and weighed.

### Novel indicator calculation

**Figure 4:**
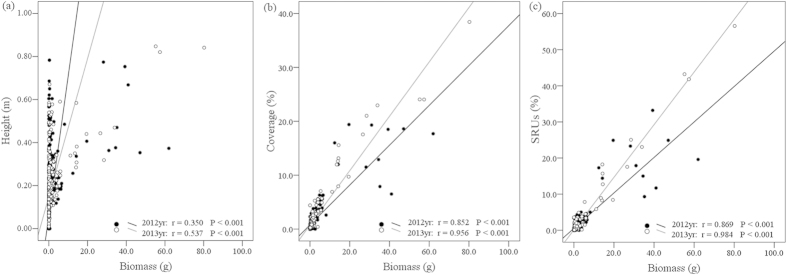
The relationship between (**a**) height and biomass (**b**) coverage and biomass (**c**) SRUs and biomass. The Pearson correlation coefficient r is shown for each pairwise combination. All correlations are significant at P < 0.05.

**Figure 5:**
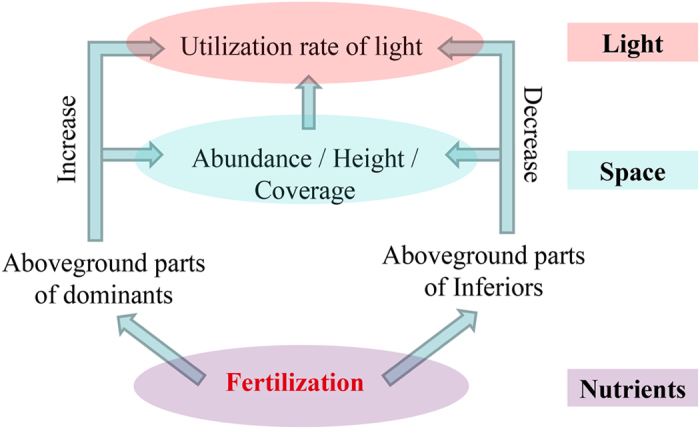
The effects of fertilisation on the plant community through multiple resources. After fertilisation, the increase in the abundance, height and coverage was considerably higher in some species, which directly affected their SRUs and subsequently indirectly affected the utilisation of light. As a consequence, these species became dominant, and other species were suppressed or died. Note that fertilisation can affect the utilisation of other resources.

**Figure 6:**
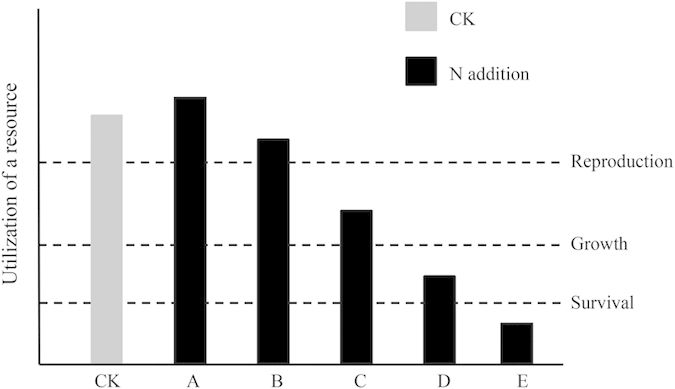
A simple model reflected different changes of particular resource utilisation after fertilisation. Three hypothetical lines drawn from the bottom upward represent the survival, growth and reproduction levels, respectively. The letters under horizontal abscissa (**A**–**E**) r**e**present five kinds of changes after fertilisation.

We calculated the theoretical volume of each species in the quadrat using a volume formula (V_i_ = h_i_ · S_i_; h_i_ = plant height of species i, S_i_ = quadrat area × percent cover of species i). Plant height is the mean value of this species’ heights. Percent cover is the ground coverage percentage of this species. The theoretical volume of each species, which was defined as the space resource utilisation of species (SRUs), was used to analyze species performance. For better comparability among different treatments, the value of SRUs is converted into percentage of SRUc, and the unit of SRUs is percentage (i.e. % in Fig. 4 and Table 1). The total volume of all the species within a quadrat, which was defined as the space resource utilisation of the community (SRUc), was used to study the variation in productivity and species richness.

### Statistical analysis

The values presented are the mean ± standard error (SE) of the six replicates. Data were analyzed separately for each year. Logarithmic transformations were used when the data violated the assumptions of normality and homogeneity of variance. Correlation analyses were used to determine the correlation between pairwise combinations of four variables (i.e., plant height, percent cover, SRUs and biomass). A one-way ANOVA and LSD post-hoc test were used to determine the effect of N addition on plant height, percent cover, SRUs and biomass. Statistical analyzes were performed using SPSS 17.0 (SPSS Inc., Chicago, IL), and differences were considered significant at P < 0.05.

## Additional Information

**How to cite this article**: Zhang, P. *et al.* Space resource utilisation: a novel indicator to quantify species competitive ability for light. *Sci. Rep.* **5**, 16832; doi: 10.1038/srep16832 (2015).

## Supplementary Material

Supporting Information

## Figures and Tables

**Table 1:** The changes in the SRUs (mean ± SE) of common species in the CK, N5, N10 and N15 treatments.

Species	2012 (%)				2013 (%)			
	CK	N5	N10	N15	CK	N5	N10	N15
*Elymus nutans*	5.0 ± 1.4	**23.3 ± 5.1**	11.7 ± 8.2	**33.2 ± 6.7**	18.2 ± 4.3	**43.2 ± 3.8**	**41.8 ± 3.9**	**56.6 ± 9.3**
*Poa crymophila keng*	0.4 ± 0.3	2.1 ± 0.8	1.1 ± 0.5	4.1 ± 3.5	1.4 ± 0.7	4.5 ± 1.2	3.6 ± 1.0	4.9 ± 2.6
*Agrostis hugoniana*	2.5 ± 0.8	1.7 ± 0.9	1.2 ± 0.8	1.2 ± 0.6	2.2 ± 0.9	1.7 ± 1.0	1.2 ± 0.7	3.1 ± 1.4
*Koeleria cristata*	3.7 ± 1.6	2.0 ± 1.2	0.9 ± 0.2	1.5 ± 0.6	0.9 ± 0.4	0.5 ± 0.4	0.3 ± 0.3	2.0 ± 1.4
*Deschampsia caespitosa*	0.5 ± 0.3	0.0 ± 0.0	1.8 ± 1.3	0.5 ± 0.4	0.3 ± 0.3	0.5 ± 0.2	0.1 ± 0.1	0.2 ± 0.1
*Scirpus pumilus*	0.1 ± 0.1	0.7 ± 0.6	0.2±0.2	0.0 ± 0.0	1.3 ± 1.0	0.0 ± 0.0	0.2 ± 0.1	0.0±0.0
*Kobresia capillifolia*	24.9 ± 5.3	19.6 ± 3.2	15.0 ± 5.4	***9.3***** ± *****3.1***	25.0 ± 7.0	23.1 ± 2.4	17.6 ± 2.4	***8.4***** ± *****2.9***
*Carex atrofusca*	1.3 ± 0.6	2.5 ± 0.7	2.7 ± 0.8	2.3 ± 0.8	0.5 ± 0.5	1.4 ± 0.5	5.8 ± 4.7	3.8 ± 1.6
*Allium sikkimense*	2.8 ± 1.2	2.3 ± 0.9	4.3 ± 2.4	1.2 ± 0.4	1.2 ± 0.2	***0.4***** ± *****0.1***	***0.3***** ± *****0.1***	***0.2***** ± *****0.1***
*Anemone obtusiloba*	1.3 ± 0.3	0.9 ± 0.1	0.7 ± 0.2	0.7 ± 0.1	1.3 ± 0.3	***0.6***** ± *****0.1***	***0.6***** ± *****0.1***	***0.3***** ± *****0.2***
*Anemone trullifolia*	0.3 ± 0.2	0.7 ± 0.3	0.6 ± 0.5	0.1 ± 0.1	0.9 ± 0.4	***0.2***** ± *****0.1***	0.4 ± 0.2	***0.1***** ± *****0.0***
*Anemone rivularis*	17.3 ± 5.2	14.4 ± 4.7	24.9 ± 3.6	17.9 ± 5.9	15.7 ± 3.8	8.3 ± 2.3	12.7 ± 3.3	8.9 ± 4.2
*Delphinium kamaonense*	2.3 ± 0.9	1.4 ± 0.8	0.7 ± 0.1	0.6 ± 0.2	0.7 ± 0.4	0.2 ± 0.2	0.2 ± 0.1	0.2 ± 0.1
*Oxytropis kansuensis*	1.2 ± 0.6	1.0 ± 0.6	0.7 ± 0.5	0.2 ± 0.2	1.4 ± 0.5	***0.0***** ± *****0.0***	***0.0***** ± *****0.0***	***0.0***** ± *****0.0***
*Astragalus polycladus*	1.4 ± 0.3	0.9 ± 0.4	***0.2***** ± *****0.1***	***0.0***** ± *****0.0***	3.0 ± 1.5	***0.3***** ± *****0.2***	***0.3***** ± *****0.3***	***0.0***** ± *****0.0***
*Thermopsis lanceolata*	1.7 ± 0.8	2.4 ± 0.7	1.7 ± 1.3	2.0 ± 0.7	1.3 ± 0.4	0.6 ± 0.3	0.6 ± 0.3	***0.3***** ± *****0.2***
*Tibetia himalaica*	0.4 ± 0.2	0.1 ± 0.1	0.4 ± 0.2	0.0 ± 0.0	0.3 ± 0.1	***0.0***** ± *****0.0***	***0.1***** ± *****0.0***	***0.0***** ± *****0.0***
*Potentilla anserina*	0.7 ± 0.4	0.9 ± 0.4	1.2 ± 0.5	0.9 ± 0.5	1.0 ± 0.4	***0.2***** ± *****0.1***	***0.2***** ± *****0.1***	***0.1***** ± *****0.0***
*Potentilla fragarioides*	1.7 ± 0.9	0.7 ± 0.2	0.5 ± 0.2	0.7 ± 0.2	1.2 ± 0.3	***0.2***** ± *****0.0***	***0.4***** ± *****0.2***	***0.0***** ± *****0.0***
*Euphorbia altotibetica*	0.4 ± 0.1	0.7 ± 0.3	0.8 ± 0.2	0.5 ± 0.1	0.5 ± 0.1	***0.3***** ± *****0.1***	0.4 ± 0.1	***0.2***** ± *****0.1***
*Gentiana sino-ornata*	0.1 ± 0.1	0.2 ± 0.1	0.2 ± 0.2	0.1 ± 0.1	0.5 ± 0.3	***0.1***** ± *****0.1***	***0.0***** ± *****0.0***	***0.1***** ± *****0.1***
*Taraxacum maurocarpum*	2.9 ± 1.4	1.4 ± 0.4	2.1 ± 1.3	0.2 ± 0.1	1.5 ± 0.8	0.3 ± 0.2	0.5 ± 0.3	0.3 ± 0.2
*Aster alpinus*	3.8 ± 2.4	1.9 ± 1.2	5.0 ± 4.6	3.4 ± 2.0	1.2 ± 0.8	0.5 ± 0.4	1.8 ± ± 1.5	0.3 ± 0.2
*Saussurea stella*	2.2 ± 0.5	2.2 ± 0.5	1.8 ± 0.5	1.6 ± 0.7	2.1 ± 0.7	1.7 ± 0.4	1.8 ± 1.0	1.1 ± 0.4
*Saussurea nigrescens*	4.6 ± 1.8	3.9 ± 2.7	3.1 ± 1.0	2.9 ± 1.3	3.4 ± 1.1	1.5 ± 1.1	1.5 ± 0.4	1.0 ± 0.4
*Geranium pylzowianum*	0.2 ± 0.1	0.3 ± 0.2	1.1 ± 0.8	0.4 ± 0.3	0.2 ± 0.1	0.4 ± 0.3	0.6 ± 0.3	0.1 ± 0.1
*Pleurospermum camtschatium*	3.1 ± 0.6	3.7 ± 0.3	2.9 ± 0.9	3.1 ± 0.7	7.8 ± 3.0	3.1 ± 1.6	3.4 ± 2.1	***1.2***** ± *****0.3***
*Euphrasia pectinata*	0.5 ± 0.3	0.2 ± 0.1	2.3 ± 2.2	0.3 ± 0.2	0.8 ± 0.3	0.4 ± 0.1	***0.1***** ± *****0.1***	***0.1***** ± *****0.0***
*Cerastium arvense*	0.1 ± 0.1	0.0 ± 0.0	0.1 ± 0.1	0.0 ± 0.0	0.2 ± 0.1	0.1 ± 0.0	0.1 ± 0.0	0.1 ± 0.1

Effects of fertilisation (N5, N10, N15) compared with the CK treatment are significant at P < 0.05. Positive and negative effects are presented in bold and bold italic font, respectively.
