# Supplementary material for: Space resource utilisation: a novel indicator to quantify species competitive ability for light
Source: Sci Rep. 2015 Nov 23;5:16832. doi: 10.1038/srep16832 (PMC4655363; doi:10.1038/srep16832)
Supplement: Supporting Information [file srep16832-s1.doc]

**Supporting Information**

**Space resource utilisation: a novel indicator to quantify species competitive ability for light**

Pengfei Zhang, Xiaolong Zhou, Junyong Li, Zhi Guo, Guozhen Du*

Table S1 The changes in plant height (mean ± SE) of common species in the CK, N5, N10 and N15 treatments. Effects of fertilization (N5, N10, N15) compared with the CK treatment are significant at P < 0.05. Positive and negative effects are presented in bold and bold italic font, respectively.

Table S2 The changes in coverage (mean ± SE) of common species in the CK, N5, N10 and N15 treatments. Effects of fertilization (N5, N10, N15) compared with the CK treatment are significant at P < 0.05. Positive and negative effects are presented in bold and bold italic font, respectively.

**Table S1**

| Species 2012 (cm) 2013 (cm)  CK N5 N10 N15 CK N5 N10 N15 |
| --- |
| | *Elymus nutans* | 48.5±4.1 | **77.4±3.7** | **66.8±6.2** | **75.3±5.7** | 58.4±5.0 | **84.7±4.4** | **82.1±4.5** | **84.1±4.1** | | --- | --- | --- | --- | --- | --- | --- | --- | --- | | *Poa crymophila keng* | 38.3±1.2 | 43.8±2.2 | **60.4±5.5** | **49.7±3.2** | 45.1±1.4 | **60.0±1.1** | **57.4±2.9** | 48.5±1.9 | | *Agrostis hugoniana* | 42.9±2.5 | 42.2±4.9 | 44.8±5.4 | 48.4±1.5 | 48.7±5.0 | 51.4±4.0 | 54.0±1.8 | 49.7±1.8 | | *Koeleria cristata* | 50.3±3.1 | 51.3±4.4 | 42.6±5.5 | 54.0±3.3 | 41.5±5.3 | 50.9±3.1 | 50.2±2.7 | 53.1±3.5 | | *Deschampsia caespitosa* | 78.3±13.3 | 0.0±0.0 | 68.6±3.3 | 65.0±4.1 | 43.9±3.7 | **66.9±7.8** | **58.4±0.0** | **58.0±11.3** | | *Scirpus pumilus* | 17.0±2.0 | 20.1±2.8 | 20.2±5.7 | 28.0±0.0 | 14.4±1.2 | 16.5±3.1 | 19.1±1.6 | 10.0±0.0 | | *Kobresia capillifolia* | 35.3±3.7 | 37.3±1.9 | 37.6±2.9 | **47.0±2.5** | 32.8±2.6 | **46.9±2.0** | **44.3±1.9** | **44.0±2.8** | | *Carex atrofusca* | 21.2±2.4 | **32.4±2.4** | **33.0±3.0** | **34.6±2.4** | 17.6±2.0 | **31.2±1.4** | **33.7±1.6** | **35.6±3.2** | | *Allium sikkimense* | 26.4±1.1 | 27.7±1.5 | **34.0±2.5** | 29.3±1.6 | 27.8±1.5 | 28.8±1.3 | 31.9±2.0 | **36.1±3.7** | | *Anemone obtusiloba* | 13.8±1.5 | 13.3±1.3 | 14.4±1.5 | 16.1±1.4 | 10.7±0.7 | 13.2±0.7 | **15.1±0.8** | 13.1±1.6 | | *Anemone trullifolia* | 9.9±3.1 | 11.5±1.1 | 10.8±1.7 | 13.0±3.0 | 10.1±1.3 | 12.7±1.4 | **16.7±1.1** | 10.0±3.0 | | *Anemone rivularis* | 25.7±3.8 | 33.6±3.7 | **40.6±3.0** | **36.3±3.6** | 30.8±4.5 | 28.5±3.4 | 38.1±3.6 | 35.0±3.6 | | *Delphinium kamaonense* | 30.7±3.9 | 26.1±4.1 | 33.1±3.6 | 30.3±5.1 | 21.7±4.9 | 25.4±12.9 | 28.4±5.7 | 27.3±11.1 | | *Oxytropis kansuensis* | 17.9±2.2 | 19.4±2.1 | 20.8±2.7 | 12.8±1.7 | 14.7±2.2 | 16.2±3.0 | 17.7±0.0 | 13.5±5.2 | | *Astragalus polycladus* | 14.4±2.0 | 17.3±2.2 | 16.9±1.8 | 6.0±0.0 | 13.6±1.0 | 19.9±1.4 | 20.2±2.7 | 10.3±0.0 | | *Thermopsis lanceolata* | 19.1±1.6 | **24.0±1.5** | 22.0±0.9 | 23.0±1.8 | 21.5±1.5 | 19.3±1.8 | 20.8±1.2 | 20.2±1.3 | | *Tibetia himalaica* | 10.2±0.8 | 14.7±3.3 | 11.9±1.6 | 23.5±0.0 | 8.4±1.1 | 12.1±2.2 | 11.3±1.9 | / | | *Potentilla anserina* | 13.9±1.5 | 15.3±1.2 | 11.9±1.2 | **21.3±1.9** | 12.3±1.3 | 15.0±2.1 | 13.7±2.3 | 11.7±3.7 | | *Potentilla fragarioides* | 13.0±1.9 | 14.7±1.7 | 13.7±1.8 | 18.0±2.3 | 11.8±1.8 | 12.5±1.6 | 14.3±2.0 | 11.0±0.0 | | *Euphorbia altotibetica* | 11.5±0.7 | **15.4±1.0** | **17.4±1.6** | **21.3±1.5** | 12.9±0.9 | **16.7±1.0** | **17.6±1.0** | **17.3±1.6** | | *Gentiana sino-ornata* | 11.2±0.2 | 15.6±2.1 | 11.6±2.7 | **18.3±1.5** | 15.8±2.4 | 9.4±0.6 | 16.3±3.5 | 15.8±0.0 | | *Taraxacum maurocarpum* | 22.9±3.7 | 22.4±2.9 | 28.2±2.4 | 18.3±2.7 | 18.9±1.5 | 25.5±4.1 | 28.3±5.0 | 23.9±0.9 | | *Aster alpinus* | 13.7±1.9 | **30.9±5.6** | 20.7±4.1 | **36.0±6.2** | 14.3±0.6 | 22.6±3.8 | 22.2±2.6 | 20.1±1.4 | | *Saussurea stella* | 17.9±2.0 | 18.9±1.2 | 18.5±2.7 | **24.7±2.6** | 17.7±1.4 | **26.0±1.4** | **23.9±1.4** | **26.4±1.4** | | *Saussurea nigrescens* | 18.3±2.0 | 19.5±1.6 | 18.3±1.8 | 21.0±2.0 | 15.8±1.7 | 19.8±0.9 | 19.9±1.3 | **22.6±1.3** | | *Geranium pylzowianum* | 17.5±2.9 | 11.2±1.3 | 15.3±2.1 | 20.6±1.9 | 9.9±1.1 | **17.2±1.4** | **16.8±0.9** | **23.0±0.3** | | *Pleurospermum camtschatium* | 19.3±2.1 | 19.7±2.0 | 23.7±2.0 | 23.2±1.9 | 25.2±2.3 | 26.6±2.4 | 23.4±2.6 | 23.6±2.6 | | *Euphrasia pectinata* | 16.0±1.7 | 19.8±1.4 | 18.1±0.6 | **27.6±1.6** | 16.4±1.2 | **20.2±1.1** | 20.3±1.6 | **21.8±1.2** | | *Cerastium arvense* | 29.4±2.2 | 18.5±0.0 | 36.0±0.0 | 23.0±0.0 | 13.7±1.5 | 10.9±1.2 | 11.9±2.2 | 16.9±2.1 | |

**Table S2**

| Species 2012 (%) 2013 (%)  CK N5 N10 N15 CK N5 N10 N15 |
| --- |
| | *Elymus nutans* | 2.6±0.7 | **11.5±3.4** | 6.5±4.6 | **18.5±3.6** | 7.9±1.8 | **24.1±2.1** | **24.0±2.8** | **38.4±8.1** | | --- | --- | --- | --- | --- | --- | --- | --- | --- | | *Poa crymophila keng* | 0.3±0.2 | 1.4±0.6 | 0.7±0.3 | 2.4±1.9 | 0.9±0.5 | 3.5±0.9 | 3.0±0.8 | **5.3±2.5** | | *Agrostis hugoniana* | 1.3±0.4 | 1.2±0.7 | 0.8±0.5 | 1.1±0.5 | 1.4±0.7 | 1.5±0.9 | 1.0±0.6 | 3.3±1.3 | | *Koeleria cristata* | 2.0±0.9 | 1.3±0.8 | 0.6±0.1 | 1.2±0.5 | 0.5±0.3 | 0.5±0.4 | 0.3±0.2 | 2.0±1.3 | | *Deschampsia caespitosa* | 0.2±0.1 | 0.0±0.0 | 1.0±0.7 | 0.4±0.3 | 0.2±0.2 | 0.4±0.2 | 0.1±0.1 | 0.2±0.1 | | *Scirpus pumilus* | 0.2±0.1 | 1.0±0.8 | 0.4±0.4 | 0.1±0.1 | 1.6±1.1 | 0.1±0.1 | 0.4±0.4 | / | | *Kobresia capillifolia* | 18.6±3.8 | 17.7±3.2 | 12.9±4.3 | ***7.9±2.1*** | 21.0±5.7 | 23.0±2.1 | 17.6±1.7 | ***9.7±3.0*** | | *Carex atrofusca* | 1.6±0.7 | 2.5±0.7 | 3.1±0.8 | 3.0±1.1 | 0.9±0.8 | 1.9±0.7 | 6.9±5.1 | 5.0±1.5 | | *Allium sikkimense* | 2.6±1.1 | 2.6±0.9 | 3.8±1.9 | 1.5±0.4 | 1.2±0.3 | ***0.6±0.2*** | ***0.4±0.1*** | ***0.3±0.1*** | | *Anemone obtusiloba* | 2.3±0.5 | 2.2±0.4 | 1.6±0.5 | 1.7±0.3 | 3.1±0.7 | 2.1±0.4 | 2.0±0.5 | ***1.1±0.5*** | | *Anemone trullifolia* | 0.7±0.6 | 2.2±1.1 | 1.1±0.8 | 0.4±0.3 | 2.4±1.1 | 0.9±0.3 | 1.0±0.6 | ***0.2±0.1*** | | *Anemone rivularis* | 16.0±3.4 | 12.0±2.9 | 19.4±3.1 | 19.3±4.8 | 13.1±3.1 | 12.6±2.2 | 15.6±4.1 | 12.0±4.1 | | *Delphinium kamaonense* | 1.8±0.6 | 1.7±0.7 | 0.8±0.2 | 0.8±0.3 | 0.6±0.2 | 0.3±0.3 | 0.3±0.1 | 0.5±0.2 | | *Oxytropis kansuensis* | 1.5±0.6 | / | 0.9±0.7 | 0.6±0.4 | 2.3±0.8 | ***0.1±0.1*** | / | ***0.2±0.1*** | | *Astragalus polycladus* | 2.5±0.5 | 1.7±0.7 | 1.5±1.0 | ***0.1±0.1*** | 4.5±2.0 | ***0.7±0.4*** | ***0.5±0.4*** | ***0.1±0.1*** | | *Thermopsis lanceolata* | 2.2±1.0 | 3.1±0.7 | 2.1±1.5 | 3.4±1.1 | 1.7±0.5 | 1.3±0.6 | 1.3±0.6 | 0.8±0.4 | | *Tibetia himalaica* | 0.8±0.4 | 0.3±0.3 | 0.9±0.5 | 0.1±0.1 | 0.9±0.3 | 0.1±0.1 | 0.2±0.1 | / | | *Potentilla anserina* | 1.1±0.6 | 1.8±0.7 | 2.9±1.1 | 1.7±0.8 | 2.2±1.0 | 0.7±0.4 | 0.6±0.4 | ***0.2±0.1*** | | *Potentilla fragarioides* | 2.5±1.0 | 1.5±0.2 | 0.8±0.3 | 1.5±0.3 | 3.1±1.1 | 0.6±0.2 | 1.2±0.5 | / | | *Euphorbia altotibetica* | 0.8±0.1 | 1.3±0.4 | 1.5±0.3 | 0.9±0.2 | 1.0±0.2 | 0.7±0.2 | 0.9±0.1 | 0.7±0.2 | | *Gentiana sino-ornata* | 0.2±0.2 | 0.5±0.1 | 0.3±0.3 | 0.3±0.1 | 0.8±0.4 | 0.4±0.3 | 0.1±0.1 | 0.2±0.2 | | *Taraxacum maurocarpum* | 3.1±1.3 | 2.0±0.6 | 2.3±1.5 | 0.6±0.3 | 1.8±0.7 | 0.5±0.2 | 0.8±0.5 | 0.6±0.4 | | *Aster alpinus* | 6.3±3.5 | 2.0±1.1 | 4.9±4.3 | 3.7±2.2 | 2.4±1.7 | 1.0±0.7 | 3.0±2.4 | 1.0±0.7 | | *Saussurea stella* | 3.3±0.8 | 3.9±0.9 | 3.1±0.9 | 2.8±1.0 | 3.0±0.8 | 3.1±0.7 | 2.4±1.1 | 2.2±0.8 | | *Saussurea nigrescens* | 5.6±1.7 | 4.7±2.9 | 5.4±1.6 | 6.3±3.0 | 5.5±1.5 | 3.3±2.3 | 3.5±0.8 | 2.6±1.1 | | *Geranium pylzowianum* | 0.3±0.2 | 0.7±0.4 | 1.7±1.1 | 0.9±0.7 | 0.6±0.2 | 1.1±0.9 | 1.6±0.7 | 0.2±0.2 | | *Pleurospermum camtschatium* | 3.9±0.6 | **6.2±0.7** | 4.5±1.3 | 5.6±1.0 | 7.0±2.1 | 4.9±2.1 | 5.7±3.3 | 2.8±0.6 | | *Euphrasia pectinata* | 0.7±0.3 | 0.4±0.3 | 2.8±2.6 | 0.5±0.3 | 1.1±0.4 | 1.0±0.3 | ***0.2±0.2*** | ***0.2±0.1*** | | *Cerastium arvense* | 0.1±0.1 | 0.1±0.1 | 0.1±0.1 | 0.1±0.1 | 0.5±0.2 | 0.3±0.2 | 0.2±0.1 | 0.3±0.2 | |
